# Can the Microwave Auditory Effect Be “Weaponized”?

**DOI:** 10.3389/fpubh.2021.788613

**Published:** 2021-12-23

**Authors:** Kenneth R. Foster, David C. Garrett, Marvin C. Ziskin

**Affiliations:** ^1^Department of Bioengineering, University of Pennsylvania, Philadelphia, PA, United States; ^2^Department of Medical Engineering, California Institute of Technology, Pasadena, CA, United States; ^3^Department of Radiology, Temple University Medical School, Philadelphia, PA, United States

**Keywords:** thermoacoustic sound generation, Frey effect, pulsed microwave energy, Havana syndrome, thresholds for adverse effects

## Introduction

Brief but intense pulses of radiofrequency (RF) energy can elicit auditory sensations when absorbed in the head of an individual, an effect known as the microwave auditory or “Frey effect” after the first investigator to examine the phenomenon (1). The effect is known to arise from thermoacoustically (TA)-induced acoustic waves in the head (2).

Lin has proposed that the Frey effect may be linked to unexplained health problems reported by U.S. officers in Cuba and elsewhere, the so-called Havana syndrome (3). The failure to detect microwave exposure to the affected individuals lends no support to this hypothesis, and we do not speculate about the cause of the symptoms. The question remains: whether the auditory effect can be “weaponized,” i.e., used to harass or harm an individual. For reasons of effect size and practicality this appears unlikely, but the lack of publicly available information about existing high power RF technology and uncertainties about thresholds for adverse effects does not allow full resolution of the matter.

## Theoretical Background

The theory of TA sound generation is well developed, [e.g., Gusev and Karabutov (4)]. There are two relevant time scales: the thermal diffusion time τ_th_ and a stress relaxation time τ_s_:


(1) $$\begin{array}{l} \tau_{th}=\frac{L^{2}}{\alpha },\;\tau_{s}=\frac{L}{v_{s}} \end{array}$$

These are, respectively, the time required for heat to diffuse out of a heated region, and for acoustic stress to propagate from that region. In Eq. 1, *L* is a distance characterizing the extent of heating, α is the thermal diffusivity and *v*_*s*_ is the speed of sound in the medium. For typical soft tissues and centimeter-scale heating patterns τ_th_ >> τ_s_ and effects of thermal diffusion are negligible.

We consider a pulse of a plane wave RF energy of duration τ and power density *I*_0_ (W/m^2^) incident normally on a plane tissue surface. The power deposition rate (Specific Absorption Rate or SAR in W/kg) at a distance x beneath the tissue surface is


(2) $$\begin{array}{l} SAR\left(x\right)=\frac{I_{o}T_{tr}}{\rho L}e^{-x/L} \end{array}$$

where *L* is the power deposition depth used to define τ_s_, *T*_*tr*_ is the fraction of incident power that is transmitted into the tissue and ρ is the tissue density (≈ 1,100 kg/m^3^). Relevant electrical and acoustic parameters are summarized in Table 1.

**Table 1:** Electrical and acoustic parameters for typical soft tissue^*^.

**F, GHz**	**L (m)Dry Skin**	***T_***tr***_***	**Stress confinement time **τ_s_** (μs)**	**p_**o**_ (Pa)** **(Assuming pulse fluence = 1 J/m^**2**^)**	**Maximum feasible pulse fluence consistent with stress confinement I_**0**_ **τ_s_** (J/m^**2**^) (assuming I**_o_ = ** 10 MW/m^**2**^)**	**Peak acoustic frequency from RF pulse of duration **τ_s_, ** kHz**	**Peak acoustic pressure from RF pulse at maximum feasible fluence (kPa) (dB re 20 μPa)**
1	1.9E-02	0.45	13	5	130	12	0.3 (144 dB)
3	9.4E-03	0.47	6	10	60	25	0.3 (144 dB)
6	4.1E-03	0.48	3	23	30	58	0.3 (144 dB)
10	1.9E-03	0.49	1	52	10	126	0.3 (144 dB)
30	4.3E-04	0.54	0.3	253	3	560	0.4 (145 dB)
100	1.8E-04	0.70	0.1	769	1	1,300	0.5 (147 dB)

*Based on electrical parameters for dry skin (7).

In the limit as τ < < τ_s_, the incremental pressure increase *p(x)* at distance x from the surface is (5).


(3) $$\begin{array}{l} p\left(x\right)=\Gamma \rho SAR\left(x\right)\tau \end{array}$$

where Γ is the dimensionless Grüneisen parameter


(4) $$\begin{array}{l} \Gamma =\frac{\beta v_{s}^{2}}{C_{p}}\approx 0.2, \end{array}$$

β is the volumetric thermal expansion coefficient, *C*_*p*_ is the specific heat capacity of the tissue, and v_s_ is the velocity of sound in the medium (≈1,500 m/s). In the limit as t → 0 the induced incremental pressure increases p and the incremental temperature increases ΔT at any point are proportional


(5) $$\begin{array}{l} \begin{array}{l} p=\Gamma \rho C_{p}\Delta T\\ \text{ where }\Gamma \rho C_{p}\approx \text{1 Pa/}\mu \text{K.} \end{array} \end{array}$$

As time progresses, two acoustic waves will propagate in opposite directions (away from and toward the interface). The latter wave will be reflected back into the tissue with a phase change that depends on the acoustic impedance mismatch at the interface. Closed-form solutions (6)^1^ and an intuitive description of the problem (4) are available. The net result is a wave propagating away from the interface that is either biphasic (due to a free boundary) or monophasic wave (due to a rigid boundary):


(6) $$\begin{array}{l} \begin{array}{l} p\left(t^{\prime }\right)=\;\frac{p_{0}}{2}e^{t^{\prime }-1}\;t^{\prime }\leq 1\text{ free boundary}\\ \;=-\frac{p_{0}}{2}e^{-t^{\prime }+1}\;t^{\prime }>1\; \end{array} \end{array}$$

(7) $$\begin{array}{l} \begin{array}{l} p\left(t^{\prime }\right)=\frac{p_{0}}{2}e^{t^{\prime }-1}\;t^{\prime }\leq 1\text{ rigid boundary}\\ \;=\frac{p_{0}}{2}e^{-t^{\prime }+1}\;t^{\prime }>1\\ \text{where }t^{\prime }=t/\tau_{s}\\ p_{0}=p\left(0\right). \end{array} \end{array}$$

The Fourier transforms of Eqs. 6 and 7 are


(8, 9) $$\begin{array}{c} \begin{array}{c} |p\left(\omega \right)|=\frac{p_{0}}{2}\frac{2\left(\omega \tau_{s}\right)}{{\left(\omega \tau_{s}\right)}^{2}+1}\text{ free boundary}\\ \;=\frac{p_{0}}{2}\frac{2}{{\left(\omega \tau_{s}\right)}^{2}+1}\text{ rigid boundary} \end{array} \end{array}$$

where ω is the radian frequency. Results are summarized in Table 1 assuming a typical soft tissue (7). These results were confirmed by numerical simulations (k-Wave Acoustic Simulation Toolbox in Matlab (Mathworks, Natick MA). The solution can be extended for longer pulses (τ _>_ τ_s_) but the efficiency of TA sound generation declines for pulses exceeding the stress confinement time. Non-linear effects (e.g., acoustic shock waves or photoinduced transparency) require far higher field strengths than presently considered.

In summary, a pulse of RF energy will induce acoustic transients in tissue. For short pulses the wave amplitude is determined by the absorbed energy per pulse or pulse fluence I_o_·τ, not pulse intensity I_o_ alone. Equal-energy pulses of millimeter waves (30–300 GHz) produce much larger acoustic waves than low-GHz pulses due to the shorter energy penetration depth (Table 1). The frequency spectrum of acoustic waves induced by RF pulses longer than τ_s_ will differ from Equations 8,9 and is adjustable *via* the pulsewidth.

In the head, the acoustic waves will be reflected from the skull, and excite the acoustic resonance of the skull, which has normal modes around 7–10 kHz for adult humans. The acoustic energy can elicit auditory sensations when it propagates to the cochlea, either directly or indirectly *via* bone conduction (the Frey effect).

## Thresholds For Perception and Adverse Effects

### Perception

Elder and Chou (8) and Lin (2) have reviewed the scant available data for thresholds of RF-induced auditory sensations. Reported thresholds vary widely, perhaps due to intersubject variability and variations in experimental method but generally correspond to fluences of ≈ 0.02–0.4 J/m^2^ for low-GHz pulses of tens of μs. From the present model, these thresholds correspond to peak acoustic pressures within the head in the range of 0.1–3 Pa for RF pulses at low-GHz frequencies.

In recent years, very high powered (gigawatt) pulsed microwave generators have been developed from low-GHz through mm-wave frequencies, many in classified defense projects. Dagro et al. (9) simulated TA waves induced in an anatomically detailed model of the body by a 5 μs pulse at 1 GHz pulse and incident power density of 10 MW/m^2^ (50 J/m^2^ pulse fluence). Dagro considered that to be “a reasonable upper limit given the publicly available literature on [high powered microwaves].” The peak acoustic pressure at any point in the brain was 10 kPa, well above that predicted by the present 1D model, which is associated with a relatively high SAR in the ventricles. These peak pressures were found in small, localized regions of brain tissue and were very brief in duration.

### Adverse Effects

The thresholds for adverse effects from such exposures can only be guessed due to lack of data. Lin (3) suggested a “tissue-injuring level” of 20 Pa for intracranial pressures based on a conventionally accepted threshold of 120 dB re 20 μPa for noise-induced hearing loss due to damage to hair cells in the cochlea. Lubner et al. described a variety of audiovestibular symptoms from ultrasound exposures above 20 kHz, for example “complaints of fatigue, buzzing, nausea, and headaches” in workers from an ultrasonic cleaning bath (115 dB at 40 kHz), with “mixed conclusions” about permanent audiovestibular damage from ultrasound exposures (10). Peak acoustic pressures shown in Table 1 far exceed these levels, but differences in exposures are considerable. In particular, TA-induced pressure waves are generated in tissues near the body surface, as opposed to ultrasound incident on the head. Dagro et al. compared the peak acoustic pressures to tensile pressures seen in typical head impacts in professional (American) football players but differences in exposure time and volume of brain tissue exposed at the highest pressure levels make such comparisons difficult to interpret.

Thresholds for ultrasonic damage to brain tissue are far higher. For example, “low energy” ultrasound is clinically used for pain relief (neuromodulation) without significant reported adverse effects in patients (11). Exposure levels to selected regions of the brain typically involve peak sound pressures above 100 kPa (194 dB) at 250–500 kHz (12).

The above discussion suggests that interactions with the audiovestibular system are likely to produce adverse but possibly reversible effects at far lower exposures than damage to brain tissue itself.

## Discussion and Conclusion

We consider whether the Frey effect could be “weaponized.” Existing microwave systems can produce pulses with sufficient fluence to induce unexpected and perhaps frightening auditory sensations, but the equipment is large and would be very obvious.

For example, the (now obsolete) AN/FPS-67B radar system generates 6 μs pulses at 1.3 GHz with a peak transmitted power of 1.9 MW (11 J per pulse). An engineer described to one of the present authors^2^ “obvious and distracting but not distressing” auditory responses while located in the main beam and 45 m from the antenna. The peak RF field strength at his location was 4.6 kV/m with a pulse fluence of ≈ 0.3 J/m^2^, which is close to the threshold for inducing auditory responses (RF exposures were well below safety limits, which are expressed in terms of time-averaged exposures). The large antenna size (37 by 15 m) and probable electromagnetic interference from the pulses would make the presence of such a transmitter very obvious.

High-frequency microwaves, in particular mm-waves (30–300 GHz) have characteristics that make them more suitable for “stealth” (not noticed) attacks. Millimeter waves cause less (or no) interference to ordinary electronics and cannot be detected with ordinary RF survey meters; the equipment is smaller and can conceivably be located much closer to the target (allowing higher exposure levels than those considered by Dagro et al.). Pulses of millimeter waves of a given fluence will induce much stronger TA acoustic waves than those at lower frequency (Table 1), but this is offset by the much shallower energy penetration depth and strong attenuation by the skull. Whether mm-wave transmitters exist that are capable of producing the extreme pulses considered here is not publicly known nor is there any evidence available to us that they played a role in the Havana incidents.

We conclude that acoustic waves induced in the brain at the “reasonable upper limit” exposures described by Dagro et al. are likely to fall short of thresholds for damaging the brain, although they conceivably could produce unpleasant audiovestibular disturbances and/or auditory responses, depending on the RF pulse duration and repetition rate. In any event, the capabilities of high-powered microwave sources remain shrouded in classified research programs and thresholds for adverse effects are poorly defined. There are easier ways to harass or harm an adversary and using directed energy weapons against people might be ill-advised for a variety of other reasons as well.

## Author Contributions

All authors listed have made a substantial, direct, and intellectual contribution to the work and approved it for publication.

## Conflict of Interest

KF and MZ have received minor research funding from an industry group (Mobile; Wireless Forum) for unrelated work. The remaining author declares that the research was conducted in the absence of any commercial or financial relationships that could be construed as a potential conflict of interest.

## Publisher's Note

All claims expressed in this article are solely those of the authors and do not necessarily represent those of their affiliated organizations, or those of the publisher, the editors and the reviewers. Any product that may be evaluated in this article, or claim that may be made by its manufacturer, is not guaranteed or endorsed by the publisher.

## Footnotes

^1^Equation 37 of (6) has an extraneous factor of 2 in the exponential.

^2^R. Weller, personal communication, 1 Oct. 2021.
